# Direct force measurement of microscopic droplets pulled along soft surfaces

**DOI:** 10.1038/s41467-022-31910-3

**Published:** 2022-07-30

**Authors:** Hamza K. Khattak, Stefan Karpitschka, Jacco H. Snoeijer, Kari Dalnoki-Veress

**Affiliations:** 1 Department of Physics and Astronomy, McMaster University, 1280 Main Street West, Hamilton, Ontario L8S 4M1 Canada; 2 Max Planck Institute for Dynamics and Self-Organization, 37077 Göttingen, Germany; 3 Physics of Fluids Group, Mesa+ Institute, University of Twente, 7500 AE Enschede, The Netherlands; 4 UMR CNRS Gulliver 7083, ESPCI Paris, PSL Research University, 75005 Paris, France

**Keywords:** Fluid dynamics, Wetting

## Abstract

When a droplet is placed on a soft surface, surface tension deforms the substrate, creating a capillary ridge. We study how the motion of the ridge dissipates energy in microscopic droplets. Using a micropipette based method, we are able to simultaneously image and measure forces on a microscopic droplet moving at a constant speed along a soft film supported on a rigid substrate. Changing the thickness of the thin film tunes the effective stiffness of the substrate. Thus we can control the ridge size without altering the surface chemistry. We find that the dissipation depends strongly on the film thickness, decreasing monotonically as effective stiffness increases. This monotonic trend is beyond the realm of small deformation theory, but can be explained with a simple scaling analysis.

## Abstract (web summary)

Elastic deformation of soft substrates occurs upon wetting, yet it is challenging to follow its dynamics at a microscale. Khattak et al. show that the force required to pull a droplet along a soft surface decreases monotonically as the film thickness decreases and explain the phenomenon using a scaling analysis.

## Introduction

Wetting is a phenomenon ubiquitous in both nature and industry, ranging from dew collection in cacti^1^ to flexible printable electronics^2^. While research on droplet-surface interactions has been an active field of research for decades, much of the work has focussed on droplets on a stiff substrate, where surface tension forces are unable to deform the substrate beyond the atomic scale^3^. On stiff substrates, it is reasonable to use the classical Young-Dupré equation for a droplet at equilibrium, and ignore any mechanical effect the droplet has on the substrate^4^. In contrast, the elastic deformation of a substrate due to surface tension is the defining feature of droplet/soft-substrate interactions. With recent advancements in soft materials and experimental methods it has been possible to study the rich physics that elastocapillarity brings^5,6^. For example, droplets may interact with each other through a substrate^7,8^ or sense the tension in the underlying substrate^9^.

In liquid/soft-substrate systems, the deformation caused by surface tension forces at the edge of a droplet is known as a capillary ridge. The size of this ridge will depend on a balance between the surface tension of the liquid droplet, *γ,* and the film modulus, *E*, and is characterized by the elastocapillary length *l*_e_ = *γ*/*E* (see Supplementary Note 1). Starting with the earlier work by Shanahan, Long, and co-workers^10–13^, more recently there have been great efforts to understand the statics and dynamics of this capillary ridge^14–23^. These efforts include direct imaging of the ridge^23,24^ and studies into how ridge shape depends on substrate mechanical properties^25^. It has been found that the ridge shape changes when a droplet is in motion^26^ and that a soft surface slows the motion of a droplet^13^. Although the capillary deformations in these cases are generally micrometric^24,27^, measurements of dynamics often rely on macroscopic liquid phases. For example, the gravitational pull on a droplet may be used as driving force^28–30^. In contrast, in the work by Gao and co-workers, direct lateral friction force was measured as droplets were pulled along hard surfaces, with the remarkable observation of static and kinetic friction in analogy with solid-solid friction^31^. Studying at the microscale allows for control of system parameters, including geometry and is of interest in applications such as microfluidics and fog collection where droplets are micrometric^32^.

**Fig. 1:**
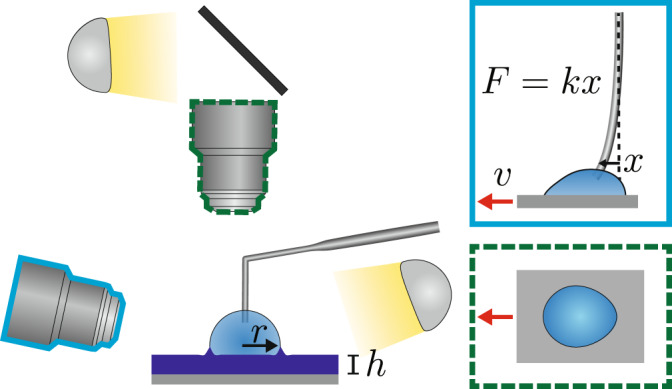
Experimental apparatus. Schematic of the apparatus used to measure the geometry and forces acting on droplets in motion. The film thickness, *h*, and size of droplet (radius, *r*) are set prior to commencing an experiment. The droplet is imaged simultaneously in two axes (schematic images from each axis included to the right). The substrate is translated at speed *v* to produce relative motion of the droplet, which is held in place by a thin micropipette. Force measurement is obtained through the deflection of the micropipette as indicated in the side axis schematic.

Here, we demonstrate a method to directly measure the force as micrometric droplets are translated relative to a substrate as shown schematically in Fig. 1. The substrates used consist of a thin soft elastomeric film with thickness *h*, supported on a rigid substrate. The advantage is that by changing the film thickness, we can tune the effective stiffness of the substrate without altering the surface chemistry. Furthermore, knowing the force *F*, required to drag a droplet at some speed *v*, we obtain the dissipated energy ${{{{{{{{\mathcal{P}}}}}}}}}_{{{{{{{{\rm{diss}}}}}}}}}$, as a function of the effective stiffness of the substrate directly. One might expect two regimes as the elastomer film thickness is decreased. First, for thick films with *h* ≫ *l*_e_, the substrate is soft, and the dissipation associated with forming and moving the capillary ridge is high and unaffected by *h*. Second, in the case of thin films with *h* ≲ *l*_e_, the capillary ridge size decreases because of the influence of the rigid substrate, which affects the dissipation. Our experiments demonstrate that the force associated with pulling a droplet along a surface is found to depend strongly on the film thickness, decreasing monotonically as *h* → 0. This monotonic trend is beyond the realm of small deformation theory but can be explained with a simple scaling analysis.

## Results and Discussion

### Sample properties and preparation

With microscale droplets the sample preparation methods are especially important. Since forces and volumes are small, effects like evaporation or sample impurities have a large effect. Furthermore, the substrate preparation is critical as the presence of uncrosslinked chains, a common issue with soft elastomeric systems, makes interpretation of the results difficult^23,29^. Elastomeric poly(dimethylsiloxane) (PDMS) substrates were prepared with various thicknesses on a Si substrate (see the Methods section for details). Upon curing of the PDMS films, some chains remain uncrosslinked. These uncrosslinked chains can significantly modify the substrate droplet-interaction dynamics as they swell the crosslinked network^23,29^. In the experiments, care was taken to remove any uncrosslinked chains using a washing procedure. After preparation of the elastomeric substrates, the samples were found to be uniform and smooth (r.m.s. roughness ≈ 1 nm), as measured with atomic force microscopy. The elastic modulus was measured to be ~500 kPa (see Supplementary Note 2 for further details on PDMS substrates).

To inhibit evaporation, droplets were prepared from an ionic liquid. The liquid has a surface tension of 40.3 mNm^−1^^33^ and we have *l*_e_ ~ 100 nm, where we have used the Young’s modulus of the substrate and the surface tension of the ionic liquid to define this characteristic experimental length scale (see Supplementary Note 1).

### Measuring the dissipation

The measurement apparatus is shown schematically in Fig. 1 and similar to that used by Backholm and co-workers^34^ to probe friction forces of droplets on superhydrophobic etched silicon surfaces. We aspirate a droplet onto a substrate, which can be translated using a motorised stage. The typical radius for the droplets is *r* ~ 100 μm, which is much greater than both *l*_e_ and *h*. A micropipette is inserted into the droplet, which holds the droplet in place as the substrate is translated back and forth. The micropipette is a long (~1 cm), thin (diameter ~10 μm), and flexible cantilever that acts as a linear force transducer (see Supplemental Methods 2 for further details). With calibration, the deflection of this micropipette can be converted into a force as low as 10 pN^35–37^. It is possible to image the droplet simultaneously from the top and side while the substrate is translated at a constant speed ranging from 0.1 μm/s to 10 μm/s. The maximum speed was set to 10 μm/s which enabled reliable force measurements for all dissipation regimes and droplet sizes.

**Fig. 2:**
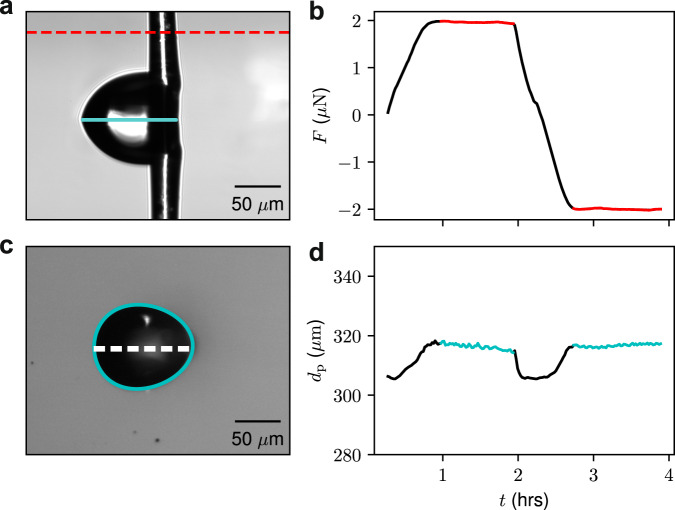
Force and perimeter measurements. **a** Side view of a droplet moving with a speed of *v* = 1 μm s^−1^. In the image shown, the substrate is moving to the left, so that the droplet motion is towards the right with respect to the substrate. The droplet equilibrium contact angle is ~80^∘^. The bottom half of the image is the reflection of the droplet and pipette in the substrate (reflection plane indicated within drop). The dashed line is the location where the force is obtained from the micropipette deflection. **b** The force required to maintain the droplet in a fixed position as the substrate is translated back and forth as a function of time. The force for a set speed, *v*, is taken from the plateau values indicated in red. **c** A top-down view of the same droplet at the same moment in time with the axis of symmetry indicated. **d** Measured perimeter as a function of time obtained from the top view. See Supplementary Movie 1 for sample experiment movie.

In Fig. 2a we show a side view of the droplet and its reflection (the reflection is the result of the microscope being slightly tilted with respect to the plane of the substrate). The side view images also provide the deflection of the pipette, and hence the force required to drag a droplet along a surface as shown in the plot of the force as a function of time in Fig. 2b. The top view of the droplet shown in Fig. 2c, is used to find the length of the droplet contact line shown in 2d. We define the radius as *r* = *d*_p_/2*π,* where *d*_p_ is the droplet perimeter since the droplets are not necessarily circular when in motion from the top view.

In a typical experiment, we drag the droplet in one direction at a set speed, *v*, and then return it to the original location at the same speed, which is repeated three times. For both force and perimeter, we see that a plateau is reached over which these quantities represent steady-state values (see Supplementary Note 3 for additional analysis details and Supplementary Movie 1 for a sample movie). Since the droplet moves at constant speed for a given plateau, the dissipation, ${{{{{{{{\mathcal{P}}}}}}}}}_{{{{{{{{\rm{diss}}}}}}}}}$, is simply given by ${{{{{\mathcal{P}}}}}}_{{{{{\rm{diss}}}}}}=vF$.

### Theoretical model and comparison to experiments

**Fig. 3:**
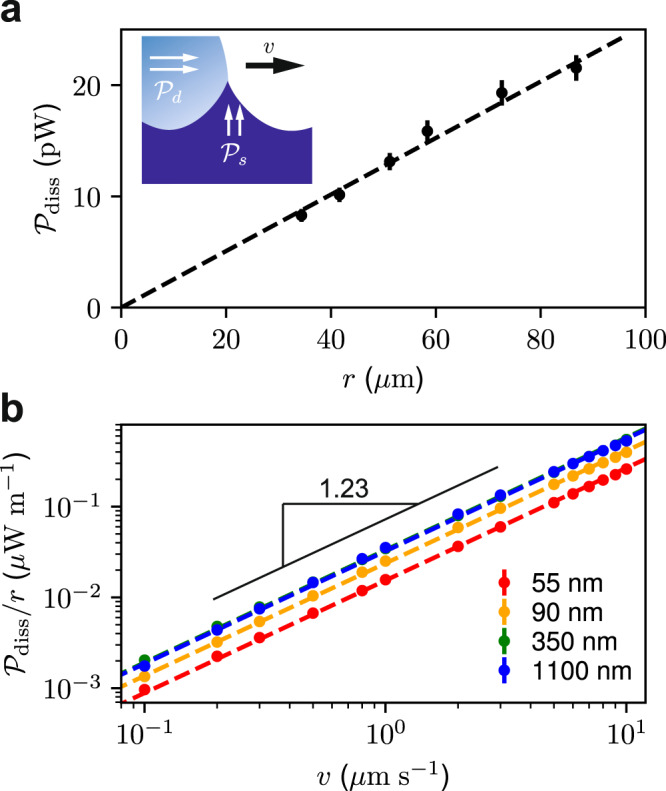
Dissipation with changing droplet size and speed. **a** Plot of the dissipation as a function of the droplet radius for a droplet moving at *v* = 10 μm s^−1^ on a substrate with *h* = (34 ± 1)nm. We calculate radius from a top view perimeter contour (*r* ≡ *d*_p_/2*π*, where *d*_p_ is the droplet perimeter) Inset: schematic of dissipation sources as ridge moves. **b** Plot of dissipation per unit length vs droplet speed for several film thicknesses. Each curve includes speeds from 0.1 μm s^−1^ to 10 μm s^−1^. Error bars for both panels are calculated using standard deviations for triplicate runs.

With our steady state force measurements, we can now develop a model for droplet dissipation following^11,13,28,38^. We expect two sources of dissipation: i) viscous dissipation in the droplet, ${{{{{{{{\mathcal{P}}}}}}}}}_{{{{{{{{\rm{d}}}}}}}}}$, due to fluid flow, and ii) dissipation in the capillary ridge of the elastic substrate, ${{{{{{{{\mathcal{P}}}}}}}}}_{{{{{{{{\rm{s}}}}}}}}}$, which is a viscoelastic material in motion (Fig. 3a inset). Thus the total energy dissipated can be written as:

1 $${{{{{\mathcal{P}}}}}}_{{{{{\rm{diss}}}}}}={{{{{\mathcal{P}}}}}}_{{{{{\rm{s}}}}}}+{{{{{{{{\mathcal{P}}}}}}}}}_{{{{{{{{\rm{d}}}}}}}}}.$$

In our system, the viscosity of the substrate prior to curing (~7000 cP) is orders of magnitudes higher than that of the droplet (~20 cP)^39^ and we can then assume the ${{{{{{{{\mathcal{P}}}}}}}}}_{{{{{{{{\rm{d}}}}}}}}}$ term can be ignored. This assumption is consistent with the work of Carré and Shanahan who showed that on soft rubbers the kinetics of droplet spreading was independent of the droplet viscosity^11^. Next we take a closer look at the substrate dissipation term. We expect the dissipation in the substrate to be associated with the capillary ridge deformation of the substrate (Fig. 3a inset). In our case, *l*_e_ ~ 100 nm, a lengthscale much smaller than the size of the droplet, *r* ~ 100 μm. We note from previous research^11,13,28,38^ that, at a scaling level, we can write:

2 $${{{{{{{{\mathcal{P}}}}}}}}}_{{{{{{{{\rm{diss}}}}}}}}}\propto vr\gamma {{\Delta }},$$

where *γ*Δ represents the force per-unit-contact-line required to deform the contact line away from equilibrium. The quantity, Δ, is dimensionless, and is rate dependent, and dependent on material properties, including the stiffness of the substrate^10–12^. While the force per-unit-contact-line depends on the material properties of the system, in this work, we focus on the droplet speed, and the thickness-dependent substrate stiffness.

The various dependencies of the dissipation can be disentangled as follows. First of all, according the the Chasset-Thirion model^40^, the viscous losses will depend on the velocity as a power-law^13^: ${(v/{v}_{0})}^{m}$; where *m* is empirical and typically varies from 0.2 to 0.7, and *v*_0_ is a characteristic substrate velocity which relates the elasto-capillary length to a characteristic time of the soft substrate *v*_0_ = *l*_e_/*τ*. Secondly, we expect the dissipation to scale with the drop size *r*, since dissipation is proportional to the length of the contact line. Hence we write Eq. (2) as:

3 $${{{{{\mathcal{P}}}}}}_{{{{{\rm{diss}}}}}}=vr \gamma \beta (h){\left(\frac{v}{{v}_{0}}\right)}^{m}.$$

Here we introduced a dimensionless dissipation parameter, *β*(*h*), that captures the sought-after thickness dependence of the effective stiffness, and which is the central object of this study.

We first verify that the proposed scaling with drop size is adequate, despite the fact that dissipation is not uniform along the contact line. Indeed, a plot of ${{{{{{{{\mathcal{P}}}}}}}}}_{{{{{{{{\rm{diss}}}}}}}}}$ as a function of *r* reveals the proposed linear relationship (Fig. 3a). We can now explore the parameters that affect the dissipation given in Eq. (3).

**Fig. 4:**
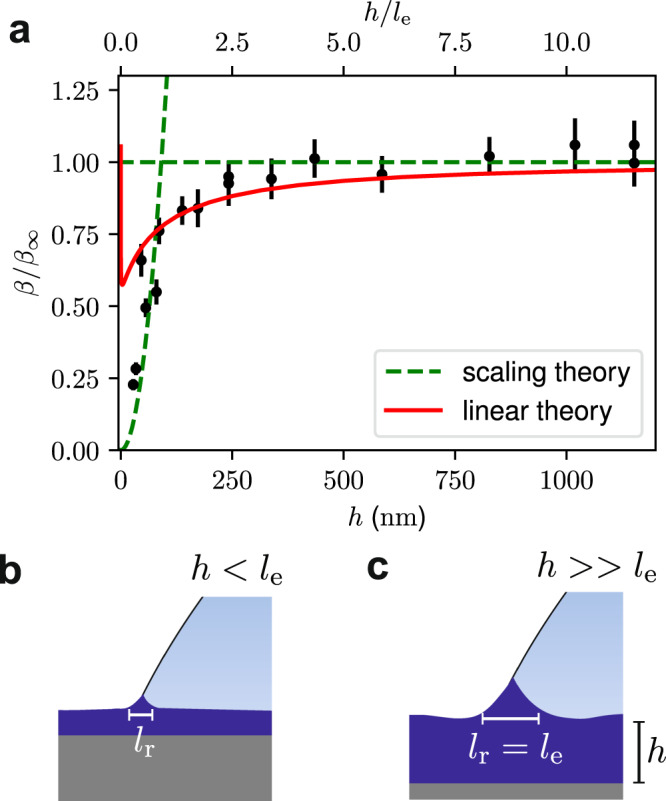
Substrate thickness effect on dissipation. **a** Plot of *β*, the dimensionless dissipation parameter, normalized by the bulk dissipation parameter, *β*_*∞*_, as a function of the soft substrate layer thickness. The dashed lines corresponds to a fit of the scaling model in the thin film and thick film regimes, while the solid line corresponds to the linear theory. Error bars are calculated from the standard error of fit to equation (3) of each experimental run. **b** and **c** illustrate the two extremes of the scaling theory for a thin substrate film, where the rigid underlying Si substrate contributes to the effective stiffness of the substrate, and the case of a thick substrate where the surface tension induced deformation is much smaller than the thickness of the film.

Since the force is measured directly, the dissipation, ${{{{{{{{\mathcal{P}}}}}}}}}_{{{{{{{{\rm{diss}}}}}}}}}=vF$, normalized by the droplet-size, ${{{{{{{{\mathcal{P}}}}}}}}}_{{{{{{{{\rm{diss}}}}}}}}}/r$ is obtained as a function of droplet speed over two orders of magnitude from 0.1 μm/s to 10 μm. As expected from Eq. (3), we observe a clear power-law response as shown in Fig. 3b. When averaged over all experiments, *m* = 0.23 ± 3, and we find no correlation of *m* with film thickness ranging from 30 nm to 1200 nm. However, it is clear from Fig. 3b that there is a dependence of the force needed to drag a droplet on the film thickness: as *h* increases, so does the force, pointing to an increase of *β* as defined in Eq. (3). Specifically, in Fig. 3b, *β* increases with thicknesses from 90 nm to 350 nm, but there is no significant change in the dissipation when comparing the data for a substrate thickness of 350 nm to 1100 nm. The thickness-dependence of the dissipation in all our measurements is summarised in Fig. 4a, where we plot the dissipation parameter *β*, normalized by the bulk dissipation parameter *β*_*∞*_, as a function of film thickness *h*. Indeed, one observes an increase of *β* with thickness, followed by a saturation at large *h*. The cross-over between the two regimes appears close the elastocapillary length *l*_e_ = 100 nm, suggesting that the transition is governed by the ratio *h*/*l*_e_.

We now wish to explain the thickness dependence of the dissipation. The results are first compared to the common framework for droplet motion over soft surfaces, which is based on the linear viscoelastic response in conjunction with small substrate deformations. Under these assumptions a closed form expression for *β*(*h*) can be obtained^38,41^ (see Supplementary Note 4). The linear theory is shown in Fig. 4a (red solid line). The result captures well the behaviour at large thickness, including the saturation, using *l*_e_ ≈ 100 nm. However, it is clear that the linear theory not only fails to describe the experiment for small *h*, but even predicts a divergence of the dissipation, which is not observed in the experiments. This breakdown can be attributed to the emergence of large deformations. Specifically, the linear theory overestimates the size of the wetting ridge as *h* → 0, which is in the linear theory ${l}_{{{{{{{{\rm{r}}}}}}}}} \sim {({l}_{{{{{{{{\rm{e}}}}}}}}}{h}^{3})}^{1/4}$^28^ (see Supplementary Note 4). Comparing such a ridge height with the thickness, this would imply a strain $\sim {({l}_{{{{{{{{\rm{e}}}}}}}}}/h)}^{1/4}$ that tends to diverge at small *h*. Clearly, the deformations encountered at small thicknesses lie outside the realm of the linear description.

From these observations we propose a simple model to explain the dependence of *β*(*h*), which is illustrated schematically in Fig. 4b, c. Since the dissipation (per unit contact line) takes place over the cross-section ${l}_{{{{{{{{\rm{r}}}}}}}}}^{2}$, one expects the dimensionless dissipation parameter to scale as $\beta \sim {({l}_{{{{{{{{\rm{r}}}}}}}}}/{l}_{{{{{{{{\rm{e}}}}}}}}})}^{2}$. On a thick substrate, the ridge is not influenced by the rigid silicon substrate, and one expects *l*_r_ ~ *l*_e_, as predicted by the linear theory. This leads to the saturation at large thickness as seen in Fig. 4a. In contrast, at small thickness the ridge will sense the stiff silicon substrate underneath. Here, linear theory eventually fails as it predicts large strains ~*l*_e_/*h* for *h* ≲ *l*_e_. Thus we hypothesize a gradual transition into a regime of strain saturation, based on geometric and/or material non-linearities that are known to arise at large strains. At saturated (constant) strain, the ridge scales with the thickness and *l*_r_ ~ *h*. Combining these two regimes we have:

4 $$\beta (h) \sim {\left(\frac{{l}_{{{{{{{{\rm{r}}}}}}}}}}{{l}_{{{{{{{{\rm{e}}}}}}}}}}\right)}^{2} \sim \left\{\begin{array}{ll}1 &{{{\mathrm{if}}}} \,\,h \, > \, {l}_{{{{{{{{\rm{e}}}}}}}}}\\ {\left(\frac{h}{{l}_{{{{{{{{\rm{e}}}}}}}}}}\right)}^{2} &{{{\mathrm{if}}}} \,\,h \, < \, {l}_{{{{{{{{\rm{e}}}}}}}}}\end{array}\right..$$

If we plot the normalised dissipation parameter *β* as a function of film thickness we see good agreement with the model (Fig. 4c). Furthermore, we see a crossover at *l*_e_ ~ 100 nm, which is in good agreement with the value obtained with bulk characterisation.

**Fig. 5:**
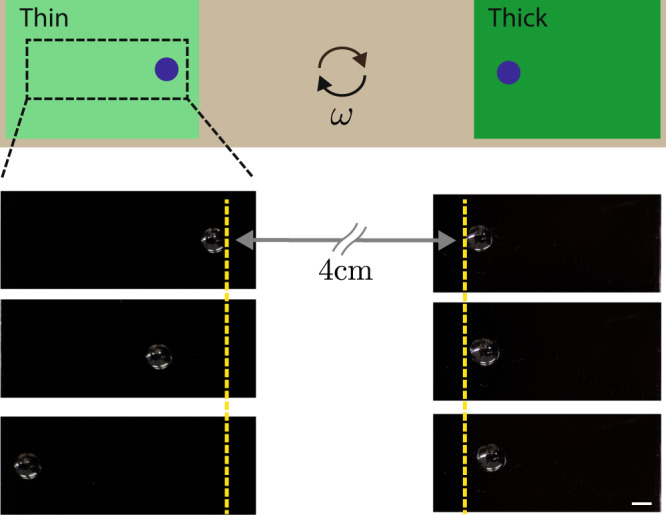
Centripetal forcing of droplets. Schematic and image sequence of centripetal forcing experiment of glycerol droplets on opposed thick (*h* > 1000 nm) and thin (*h* < 100 nm) PDMS films. The top image represents initial position of the droplets. Subsequent frames are separated by 30 s rotations on a spin coater at 200 rpm. The scale bar is 2 mm.

As a final validation, a test is carried out with millimetric droplets as follows: on a spincoater we simultaneously place an equally sized droplet on a thin substrate, and one on a thick substrate at the same distance from the centre of rotation (Fig. 5). Upon spinning, we see that while the droplets experience the same centrifugal body force, the droplet on the thin film moves further from the centre than the droplet on the thick film due to the difference in energy dissipated, consistent with Eq. (3).

### Conclusions

Our findings demonstrate a widely extendable method for studying forces in microscale droplet surface interactions. We are able to control the energy dissipation as a liquid moves along a soft substrate and find that dissipation is strongly dependent on the effective stiffness of the substrate. The results are captured by a scaling analysis that, for small thickness, highlights a breakdown of the standard “small-deformation” theory. The flexibility of our method with respect to geometry opens up immediate opportunities to study other geometries, including free-standing films and fibers. The microscale lengthscale may also make it possible to study the transition between transport regimes, including where diffusion becomes relevant. Gaining an understanding of such systems could have implications in fields ranging from microfluidics to water collection.

## Methods

### Substrate preparation

To prepare our PDMS stock mixture we combine DMS-V35 and HMS-064 (Gelest) in a stoichiometric 20:1 ratio and mix mechanically. The DMS-V35 contains vinyl terminated end groups that can bond to methylhydrosiloxane sites on HMS-064. This stock mixture is diluted in toluene from 1% to 20% wt/wt solutions appropriate for spin coating films resulting in films with thickness, *h*, ranging from 15 nm to 1500 nm. Immediately prior to spin coating, an excess of catalyst (Platinum(0)- 1,3-divinyl-1,1,3,3-tetra-methyldisiloxane, Sigma-Aldrich) is added to the dilute solution (Approximately 20 μL of 1000x diluted catalyst to 1 ml of solution). We spin coat onto ~1 cm^2^ sections of Si (University Wafer, 100 orientation) at 3500 rpm for 1 minute at maximum acceleration (Speciality Coating Systems P6000). Following spin coating, the films are cured for 4 hrs at 80 ^∘^C on a hot plate.

In order to determine an appropriate protocol, cured samples were placed on the spincoater and flooded with toluene for some time, after which the sample was spun to remove excess solvent. Measuring the thickness of the film after subsequent treatment with ellipsometry could track the loss of uncrosslinked chains as a function of cumulative exposure to toluene. As much as 30% of the as-cured sample mass is removed in the rinsing process (see Supplementary Note 2). Such examination of samples resulted in the final protocol used in the measurements presented here: three rinses on the spincoater for 60 s, followed by storing the sample in a large volume toluene bath. After 24 hrs in the toluene bath, the samples were removed, and excess solvent removed by spinning. This process was effective at removing uncrosslinked chains as measured by ellipsometry as well as through consistent contact angles in long-term experiments.

### Droplet preparation

The ionic liquid (1-ethyl-3-methylimidazolium dicyanamide, Sigma-Aldrich) was used for the droplets to avoid evaporation. These liquids have vapour pressures low enough that even microscopic droplets (~100 μm) can be studied over several days without notable evaporation^39^. The droplets can be deposited onto the PDMS substrate using a glass micropipette.

## Supplementary information

Supplementary Information

Description of Additional Supplementary Files

Supplementary Movie 1

## Data Availability

Data that support the findings of this study are available from the corresponding author (KDV) upon reasonable request. The data files are many terabytes and not hosted online.
