# Supplementary material for: Direct force measurement of microscopic droplets pulled along soft surfaces
Source: Nat Commun. 2022 Jul 30;13:4436. doi: 10.1038/s41467-022-31910-3 (PMC9338979; doi:10.1038/s41467-022-31910-3)
Supplement: Supplementary file 1 — Supplementary Information [file 41467_2022_31910_MOESM1_ESM.pdf]

Supplementary Information:
Direct force measurement of microscopic droplets pulled along soft surfaces

Hamza K. Khattak,[1] Stefan Karpitschka,[2] Jacco H. Snoeijer,[3] and Kari Dalnoki-Veress[1,4,*]

[1] *Department of Physics and Astronomy, McMaster University, 1280 Main Street West, Hamilton, Ontario, L8S 4M1, Canada*
[2] *Max Planck Institute for Dynamics and Self-Organization, 37077 Göttingen, Germany*
[3] *Physics of Fluids Group, Mesa+ Institute, University of Twente, 7500 AE Enschede, The Netherlands*
[4] *UMR CNRS Gulliver 7083, ESPCI Paris, PSL Research University, 75005 Paris, France.*

Contents:

Supplementary Note 1. Defining elastocapillary length
Supplementary Note 2. Sample preparation and characterisation
Supplementary Note 3. Measurement details
Supplementary Note 4. Linear viscoelasticity theory

SUPPLEMENTARY NOTES

Supplementary Note 1: Defining elastocapillary length

We note that the deformation normal to the substrate depends on the surface tension of the liquid, while the lateral extent of the deformation scales with the surface tension of the elastic substrate. For simplicity, here we simply characterize the scale of the capillary ridge as the ratio of the Young's modulus and the liquid surface tension.

Supplementary Note 2: Sample preparation and characterisation

The spincoating is described in the main text, we use the maximum acceleration of $\sim$ 200cycles/s^2. The exact value of the acceleration is not strongly tied to the quality of the films. We spincoat the films with the highest acceleration at 3500 rpm and select the films that are most uniform by visual inspection (colour), optical microscopy, atomic force microscopy for roughness, and we measure the resulting thickness using ellipsometry. Following spin coating, films are cured for 4 hrs at 80 °C on a hot-plate and covered to avoid any dust. Uncrosslinked chains are removed by flooding the sample while placed on the spincoater with toluene. In order to determine how much exposure to solvent was required to remove uncrosslinked chains, samples were flooded with toluene. The excess toluene was removed by spinning, the thickness measured with ellipsometry (Accurion EP4), and then the process was repeated with the same samples to obtain a plot of the thickness as a function of cumulative exposure to toluene (Supplementary Figure 1a). The final step was to place the sample in a large volume bath of toluene for 24 hrs and then again the solvent was removed by spinning. We see that for all three thicknesses as much as 30% of the film thickness is lost by removing uncrosslinked chains. In these thin films, even after exposure to toluene for 60 s the plateau (given by the 24 h measurement) thickness is reached.

For the samples used in the measurements presented in the main text, the as-cured substrates were flooded with toluene as discussed above for 60 s three times. Finally the samples were placed in a large toluene bath for 24 h, removed, and then the excess toluene was removed by spinning. As evidenced by the loss of thickness as well as through consistent contact angle measurements in long term experiments, this process is effective at removing uncrosslinked chains. Lastly we verify the microscale uniformity of the films through atomic force microscopy to ensure that they are sufficiently smooth (Supplementary Figure 1b).

To characterise bulk mechanical properties we first cure a small ∼1 cm^3 cube of our mixture at 80 °C for 24 h. This sample is then placed on a microscope slide on a standard microbalance. We compress this cube using a second slide which is translated vertically using a digital micrometer. With this method we are able to measure a force displacement curve for our cube of material. Any movement in the slide and balance itself (generally minimal) is accounted for using the same measurement without a sample (We assume the measured effective spring constant follows a simple series spring formulation: $k_{measured} = \left(\frac{1}{k} + \frac{1}{k_o}\right)^{-1}$ where k is the spring constant of the material and k_o is the no-sample spring constant). To calculate the Young's modulus we assume an incompressible material with

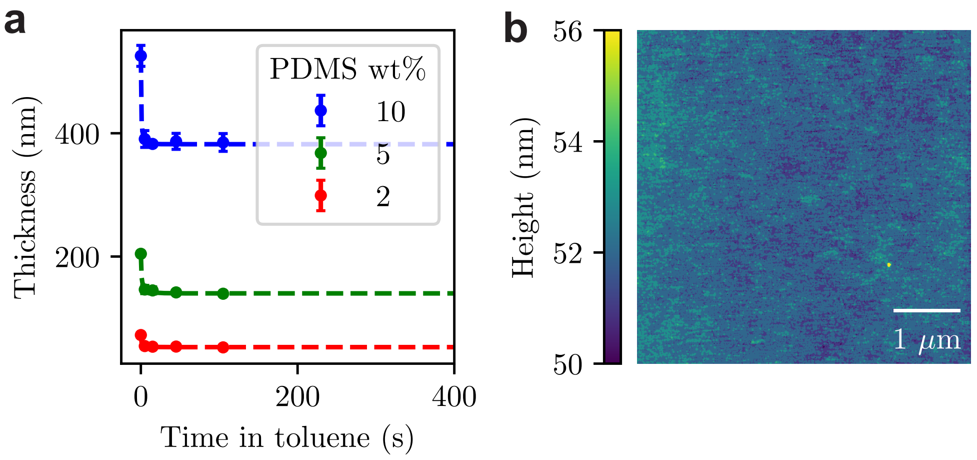

Supplementary Figure 1. **a)** Thickness as a function of time in toluene for an assortment of PDMS concentrations as measured with ellipsometry. The plateau value is obtained by taking the samples and exposing them to a further 24 hours in a large volume toluene bath. Error bars are calculated using standard deviations of triplicate samples at a given thickness. **b)** Atomic force microscopy image of a sample PDMS film (Bruker Multimode)

a Poisson's ratio of 1/2 and extract the slope of a pressure-strain plot. We determine the modulus to be ∼500 kPa.

Supplementary Note 3: Measurement details

We produce pipettes (diameter ∼10 µm, length ∼10 cm) by pulling a glass borosilicate capillary tube ($d = 1$ mm) using a heating element and constant force puller (Narishinge PN-31)(for thicker diameters, a more rudimentary flame heating with pulling using an elastic band is sufficient). To prevent any aspiration of liquid, we melt the end of the pipette using close contact to a platinum heating element. The pipette is bent so it can be mounted in a location that won't interfere with imaging. To calibrate the pipette force constant the the gravitational force from small known masses of copper wire can be compared to measured pipette deflection.

To measure pipette deflections, we use a cross-correlation analysis. We take the the intensity profile along a slice of the image containing the pipette. This intensity profile is then shifted horizontally pixel by pixel to create an array of shifted intensity profiles. These profiles are compared to a reference profile for an an unforced pipette using a standard cross-correlation definition ($c_{av}[k] = \sum_n a[n+k] * \overline{v[n]}$ where a and v are the functions to compare). By finding the peak of this cross-correlation curve using a Gaussian fit, we can find a sub-pixel value for the displacement of the pipette and with a calibrated pipette, forces as low as 10 pN can be measured [1–3].

To measure the contact patch length, we use an intensity threshold to find the edge of the droplet. We then fit an egg-like expression ($Ax+By+Cx^2+Dxy+Ey^2+Fxy^2+Gx^2y$) to the profile and use the perimeter of the egg to extract a contact patch length.

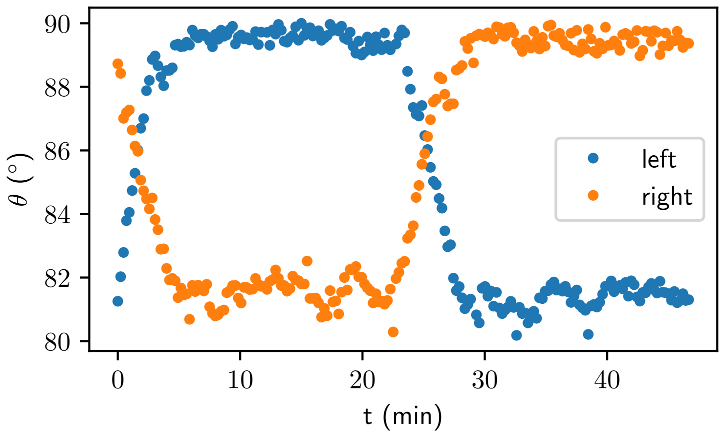

Supplementary Figure 2. Contact angle, θ vs time for a sample experiment involving a droplet dragged left and then dragged right along a soft surface (Droplet moving at $0.5\,\mu\mathrm{m\,s^{-1}}$). This measurement is done optically at the microscale and does not represent the behavior at the capillary ridge.

We perform the cross-correlation and perimeter analysis on each frame in an image series as the substrate is dragged back and forth. In both cases, interactions with dust particles can result in temporary spikes in forces and perimeter. To prevent these being included in analysis we set a threshold for a maximum allowable slope and acceleration in the force value which removes these spikes. See Supplementary Video 1 for a sample experiment and extracted force and perimeter with time.

We can also measure the contact angles of the droplets optically, (Supplementary Figure 2), however these angles do not correspond to the behavior at the nansocale contact ridge.

Supplementary Note 4: Linear viscoelastic theory

Here we recall the small-deformation theory for contact line motion, as available in the literature, but extract the thickness-dependence of the dissipation. This follows the Fourier-Transform approach of [4], and makes use of the deformation of an incompressible viscoelastic layer (undeformed thickness h) in response to a moving line force according to [5]. We assume translational symmetry along one surface direction, i.e. the 2D plane strain problem. In the co-moving frame, the substrate profile $\widetilde{\xi}(q)$, relative to the reference configuration and scaled by $\frac{3\gamma}{E}$ is

$$\widetilde{\xi}(q) = \frac{1}{\widehat{\psi}(-v\,q)/\widetilde{\mathcal{K}}(q) + \alpha_s\, q^2} \tag{1}$$

where q is the wave vector in units of h^{-1}, $\alpha_s = \frac{3\gamma_s}{E\,h}$ the elastocapillary number, defined with the solid surface tension γ_s, the static Young's modulus E and the (undeformed)

substrate thickness h. $\widehat{\psi}(\omega) = \frac{E'(\omega/\tau)+i\,E''(\omega/\tau)}{E}$ is the scaled complex Young's modulus, ω is the angular frequency in units of τ^{-1}, where τ is the relaxation time scale. v is the velocity of the moving line force, given in units of h/τ. $\widetilde{\mathcal{K}}(q)$ is the elastic Green's function.

Consistent with experiments (see main text) we use a Chasset-Thirion constitutional model,

$$\widehat{\psi}(\omega) = 1 + (i\omega)^n \tag{2}$$

with rheological exponent m and the thin-layer geometric kernel

$$\widetilde{\mathcal{K}}(q) = \frac{1}{2q}\,\frac{\sinh(2q) - 2q}{\cosh(2q) + 2q^2 + 1} \tag{3}$$

The dissipative force $\frac{\mathcal{P}_{\rm diss}}{v}$, per unit length of contact line, in units of $\frac{3\gamma^2}{E\,h}$, then reads [6, 7]

$$\frac{\mathcal{P}_{\rm diss}}{v} = \frac{1}{\pi}\int_0^\infty dq\,\frac{q\,G''(v\,q)\,\widetilde{\mathcal{K}}(q)}{\left(G'(v\,q) + \alpha_s\,q^2\,\widetilde{\mathcal{K}}(q)\right)^2 + G''(v\,q)^2} \tag{4}$$

The integrand can be expanded for small v, which essentially eliminates the velocity dependence of the ridge geometry and enables us to extract the v-dependence explicitly:

$$\frac{\mathcal{P}_{\rm diss}}{v} = \frac{v^m\,\sin m\pi/2}{\pi}\int_0^\infty dq\,\frac{\widetilde{\mathcal{K}}(q)\,q^{m+1}}{\left(1 + \alpha_s\,q^2\,\widetilde{\mathcal{K}}(q)\right)^2} \tag{5}$$

The dissipation parameter $\beta(h)$ is defined analogously to the main manuscript, up to a geometric factor, due to the plane-strain assumption in contrast to a finite-size droplet:

$$\frac{\mathcal{P}_{\rm diss}}{v} = \frac{E\,h}{3\gamma}\beta(h)\left(\frac{v}{\alpha_s}\right)^m. \tag{6}$$

Thus we obtain a closed-form expression for $\beta(h)$:

$$\beta(h) = \frac{3\gamma}{E\,h}\,\frac{\alpha_s^m\,\sin m\pi/2}{\pi}\int_0^\infty dq\,\frac{\widetilde{\mathcal{K}}(q)\,q^{m+1}}{\left(1 + \alpha_s\,q^2\,\widetilde{\mathcal{K}}(q)\right)^2} \tag{7}$$

In the limit $\alpha_s \to 0$ (the limit of thick layers), the kernel $\widetilde{\mathcal{K}}(q)$ can be replaced by its large-q-asymtote, the kernel for elastic half space, $\widetilde{\mathcal{K}}(q) \sim \frac{1}{2|q|}$, and the integral can be performed analytically [5]:

$$\beta_\infty = \frac{\gamma}{\gamma_s}\,\frac{2^{m-1}\,m}{\cos\frac{m\,\pi}{2}} \tag{8}$$

In the opposite limit, $\alpha_s \to \infty$ (very thin layers), the kernel can be replaced by its small-q-asymptote, $\widetilde{\mathcal{K}}(q) \sim \frac{q^2}{3}$. Again one can perform the integration analytically:

$$\beta\left(h \ll \frac{3\gamma_s}{E}\right) = \frac{\gamma}{\gamma_s}\,\left(3\,\alpha_s^3\right)^{m/4}\,\frac{m\,\cos\frac{m\,\pi}{4}}{8} \tag{9}$$

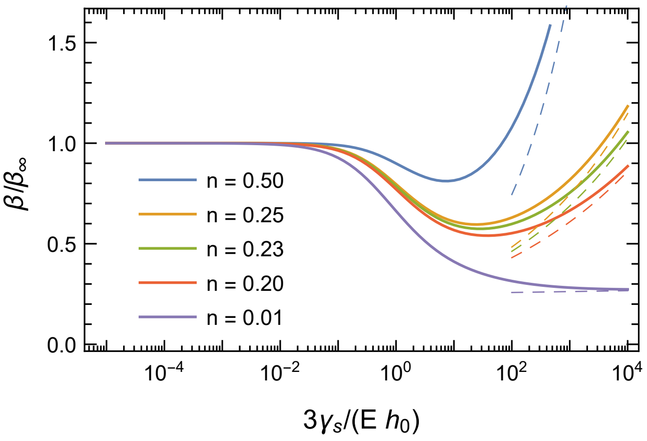

Supplementary Figure 3. Geometric dissipation factor β/β_∞, as a function of the elastocapillary number (logarithmic scale). Dashed: large-α_s-asymptote (Eq. 9).

From this expression it can be inferred that the natural velocity scale is no longer given by the elastocapillary length, but by $(3\gamma_s/E)^{1/4}h^{3/4}$, which is the lateral ridge extent [8]. Keeping velocity and elastocapillary length constant, while decreasing the layer thickness, the above expressions shows that dissipation diverges $\sim h^{-3n/4}$.

These asymptotics of the linear viscoelastic theory have been confirmed from direct numerical evaluation of the integral in Eq. 7. The result is plotted in Supplementary Figure 3, scaled by β_∞ from Eq. 8. The green line for $m = 0.23$ is used in Figure 4 of the main text.

SUPPLEMENTARY REFERENCES

* dalnoki@mcmaster.ca

[1] M. J. Colbert, A. N. Raegen, C. Fradin, and K. Dalnoki-Veress, Adhesion and membrane tension of single vesicles and living cells using a micropipette-based technique, Eur. Phys. J. E **30**, 117 (2009).

[2] M. Backholm, W. S. Ryu, and K. Dalnoki-Veress, Viscoelastic properties of the nematode Caenorhabditis elegans, a self-similar, shear-thinning worm, Proc. Natl. Acad. Sci. U. S. A. **110**, 4528 (2013).

[3] R. D. Schulman, M. Backholm, W. S. Ryu, and K. Dalnoki-Veress, Undulatory microswimming near solid boundaries, Phys. Fluids **26**, 101902 (2014).

[4] D. Long, A. Ajdari, and L. Leibler, Static and dynamic wetting properties of thin rubber films, Langmuir **12**, 5221 (1996).

[5] S. Karpitschka, S. Das, M. van Gorcum, H. Perrin, B. Andreotti, and J. H. Snoeijer, Droplets move over viscoelastic substrates by surfing a ridge, Nat. Commun. **6**, 7891 (2015).

[6] S. Karpitschka, S. Das, M. van Gorcum, H. Perrin, B. Andreotti, and J. H. Snoeijer, Soft

wetting: Models based on energy dissipation or on force balance are equivalent, Proc. Natl. Acad. Sci. U. S. A. **115**, E7233 (2018).

[7] M. Van Gorcum, S. Karpitschka, B. Andreotti, and J. H. Snoeijer, Spreading on viscoelastic solids: are contact angles selected by Neumann's law?, Soft Matter **16**, 1306 (2020), arXiv:1907.08067.

[8] M. Zhao, J. Dervaux, T. Narita, F. Lequeux, L. Limat, and M. Roché, Geometrical control of dissipation during the spreading of liquids on soft solids, Proc. Natl. Acad. Sci. U. S. A. **115**, 1748 (2018).
