# Supplementary material for: Direct force measurement of microscopic droplets pulled along soft surfaces
Source: Nat Commun. 2022 Jul 30;13:4436. doi: 10.1038/s41467-022-31910-3 (PMC9338979; doi:10.1038/s41467-022-31910-3)
Supplement: Supplementary file 2 — Description of Additional Supplementary Files [file 41467_2022_31910_MOESM2_ESM.pdf]

Description of Additional Supplementary Files

Supplementary Movie 1: Video of the experiment. Top left panel is a side view of the droplet (used to get the force). Top right panel is a top view of the droplet (used to get the perimeter). Bottom panels show the force and perimeter of the droplet as a function of time.
